# Evaluating Mediterranean Diet-Adherent, Healthy and Allergen-Free Meals Offered in Tarragona Province Restaurants (Catalonia, Spain): A Cross-Sectional Study

**DOI:** 10.3390/nu13072464

**Published:** 2021-07-19

**Authors:** Floriana Mandracchia, Elisabet Llauradó, Rosa Maria Valls, Lucia Tarro, Rosa Solà

**Affiliations:** 1 Functional Nutrition, Oxidation, and Cardiovascular Diseases Group (NFOC-Salut), Healthy Environment Chair, Facultat de Medicina i Ciències de la Salut, Universitat Rovira i Virgili, 43201 Reus, Spain; floriana.mandracchia1@urv.cat (F.M.); elisabet.llaurado@urv.cat (E.L.); rosamaria.valls@urv.cat (R.M.V.); rosa.sola@urv.cat (R.S.); 2 Institut d’Investigació Sanitària Pere Virgili (IISPV), 43204 Reus, Spain; 3 Hospital Universitari Sant Joan de Reus, 43204 Reus, Spain

**Keywords:** restaurants, healthy menu choices, food allergy, Mediterranean diet, cross-sectional

## Abstract

Restaurant meal consumption has increased substantially, but the ability of restaurants to adhere to guidelines for the Mediterranean diet, healthiness and food allergen management is a challenge. This cross-sectional study aims to assess the Mediterranean diet adherence, healthiness, nutritional quality and food allergen management of meals at restaurants in the Tarragona province (Catalonia, Spain). Primary outcomes included adherence to criteria for the Mediterranean diet (AMed) and gluten management (SMAP), nutritional quality of dishes indicated by a green traffic light rating, meal nutrient content and allergen-free options. Secondary outcomes included restaurant staff knowledge about the Mediterranean diet and food allergens. Forty-four restaurants and 297 dishes were analysed. The restaurants fulfilled an average (mean ± SD) of 5.1 ± 1.6 of 9 compulsory AMed criteria and 12.9 ± 2.8 of 18 SMAP criteria. Dishes were mainly rated green for sugar (*n* = 178/297; 59.9%) but not for energy (*n* = 23/297; 7.7%) or total fat (*n* = 18/297; 6.1%). Waiters and cooks received passing scores for food allergen knowledge (5.8 ± 1.7 and 5.5 ± 1.5 out of 10 points, respectively). Restaurants partially met the AMed and SMAP criteria. Increasing fibre and decreasing saturated fat content are necessary to improve consumers’ adherence to healthy diets. For restaurant staff, training courses should be considered to improve their food allergen management.

## 1. Introduction

The consumption of daily meals outside of the home, both in sit-down restaurants and as take-away foods, has increased in recent years among adults and children [1]. Lack of time and work commitments have been reported as the major reasons for the consumption of daily meals in restaurants and cafeterias. For example, in the region of Catalonia (Spain), out-of-home consumption occurs on average 3.5 times a week [2]. Additionally, according to a recent study of eleven European countries, eating frequently at restaurants was associated with a higher intake of energy, fat and alcohol [3], as well as with a lower consumption of fruits and vegetables, increasing the risk of weight gain, overweight and obesity [4].

The food environment is directly associated with the nutritional quality of the foods offered, so consumers’ diets differ according to the eating location; for instance, out-of-home meals consumed in the workplace setting are nutritionally healthier and more similar to home-cooked meals than restaurant and fast-food meals, as more high energy-density foods are available at these locations [5]. In recent years, in Mediterranean countries, the frequency of restaurant meal consumption has been correlated with an obesity epidemic [6]. Furthermore, it has also been observed that restaurants’ food choices are influenced by socioeconomic and demographic factors, such as financial status and population age, that impact the nutritional quality of the menu choice [7]. For instance, food poor in nutrients and high in energy is associated with lower cost and higher affordability by low-income populations and youth [8]. However, offering healthier meals [9] and reducing the price of healthier options [10] could influence consumers’ food choices, leading to an increase in the purchase and consumption of out-of-home healthier items. Similarly, the provision of nutritional information on restaurant menus has been shown to be positively related to the purchase of lower-fat and healthier meals by consumers, who are willing to spend more on healthier products if nutrient labelling is provided, and the healthier choice is more visible to them [11]. On the other hand, one of the main concerns for restaurateurs in the implementation of healthier meals and nutrient information is the loss of profit that could result from it; thus, changes in community nutritional policies and norms could support restaurants through the provision of more incentives [12] for the improvement of menus offering to include meals with better nutritional quality [7]. However, the value that restaurateurs place on community health is the most important driver to encourage healthy improvements since the lack of interest by restaurants has been found to be a major barrier in the effectiveness of interventions to promote healthy eating [13].

Additionally, eating outside of the home also represents a difficulty for people suffering from food allergies and intolerances, with 21–31% of accidental allergen ingestion occurring in restaurants [14] due to the lack of adequate training of staff on the proper prevention and management of food allergens by employees [15]. Although European Union (EU) regulation No. 1169/2011 requires food businesses to declare the presence of any of the 14 specified food allergens (peanuts, tree nuts, milk, soya, mustard, lupin, eggs, fish, molluscs, crustaceans, cereals containing gluten, sesame, celery and sulphites) in the offered foods [16], allergic consumers would like to be able to count on qualified staff and on a safer food environment [17].

Previous cross-sectional studies have assessed the energy and nutrient contents of purchased [18] and served [19,20] restaurant meals, as well as the degree of knowledge of restaurant staff about food allergen management [21,22,23] in different countries, such as Germany, the USA, the UK, Turkey and Canada, but there is still a lack of data about the nutritional quality of Spanish restaurants. However, several studies have demonstrated the high quality of Spanish olive oil in terms of its psychochemical and sensory components [24] and its benefits for human health in reducing the risk of developing chronic diseases [25,26] and that it is the most important food element for the Mediterranean diet [27].

In this context, restaurant-based cross-sectional analysis represents the first step of assessing the characteristics and nutritional quality of the meals offered in local full-service restaurants. The obtained information will provide a basis to design an intervention aimed at increasing restaurant offerings of healthier meals, as well as of allergen-free food options adapted for people with food allergies and intolerances.

Thus, the aim of this cross-sectional analysis was to assess the healthiness and nutritional quality of meals offered and their adherence to the Mediterranean diet as well as food allergen-adapted meals and their management at restaurants of the Tarragona province (Catalonia, Spain).

## 2. Materials and Methods

### 2.1. Study Design

The present cross-sectional study is about the nutritional quality of menus offered in restaurants and the availability of allergen-free dishes of Tarragona province restaurants. It includes the baseline data of the Healthy Meals Randomized Controlled Trial (RCT), which is a multicomponent intervention applied to restaurants and their staff, including training, menu nutritional quality analysis and identification of food allergens, to promote healthier meals for each member of a family and improved management of food allergens, and to satisfy customers with specific needs (food allergies and intolerances). It was carried out from September 2019 to March 2021, before and during the SARS-CoV-2 pandemic. This study is part of a European funded project called PECT-TurisTIC en Familia, through which the University Rovira i Virgili (Tarragona, Spain) has led the “Healthy Meals” operation, one of the twelve operations included in the project. The study was conducted in accordance with the Declaration of Helsinki [28], and the protocol was approved by the Ethics Committee of the Institut d’Investigació Sanitaria Pere Virgili (ref CEIM: 179/2018). The trial was registered in 2019 at the international registry of clinical trials (ClinicalTrials.gov) [29] with the project identification code NCT03826576. All restaurant owners gave their informed consent for inclusion before they participated in the study. Moreover, to ensure the study quality, the present study followed the STrengthening the Reporting of OBservational studies in Epidemiology (STROBE) criteria [30] for observational cross-sectional studies (Table S1).

### 2.2. Study Population and Setting

The study population consisted of full-service restaurants offering traditional and Mediterranean cuisine that were recruited from the province of Tarragona (Spain). Restaurants were searched, considering the population density of the different counties of the Tarragona province [31], through the tourism offices of each participating town and online restaurant databases, such as TripAdvisor [32]. The study researchers visited the restaurants and explained the study to the owner/responsible party of each restaurant to obtain informed consent. Then, the researchers verified whether the restaurants met the inclusion criteria: (1) being a full-service restaurant; (2) having a minimum of 5 serviced tables; (3) offering Mediterranean/traditional/local cuisine; (4) having technical details of the recipe for each dish, including ingredients and cooking details; (5) being willing to share food product information with the research team; and (6) the owner signing an informed consent form for participation in the study. On the other hand, restaurants were excluded if they (1) were ethnic or fast-food restaurants; (2) had fewer than 5 serviced tables; or (3) had not yet received the Mediterranean diet (AMed) certification.

Furthermore, restaurant owners had to (1) have a minimum of one year of experience and (2) be available to continue working during the one-year intervention. Not fulfilling any of the above inclusion criteria led to exclusion from the study.

### 2.3. Outcomes and Data Collection

The primary outcomes included the following:

(1) number of compulsory AMed criteria, which must be fulfilled by restaurants to obtain a certification that the restaurant offers a Mediterranean diet (described below), as determined through face-to-face interviews with the restaurant owner;
(2) number of SMAP criteria fulfilled by restaurants, as assessed through face-to-face interviews with the restaurant owner to determine the potential for obtaining the corresponding certification;
(3) number of dishes per restaurant with a green rating in the traffic light rating system on the Healthy Meals app for the evaluated nutrients (energy, carbohydrates, sugar, fat, saturated fat, protein, sodium and fibre), which indicates good nutritional quality, according to the analysis of the recipes of the dishes, including the ingredients, weights and cooking details;
(4) nutrient content of the restaurants’ meals (kcal of energy; grams of carbohydrates, sugar, fat, saturated fat, protein and fibre; micrograms of sodium), assessed by the Healthy Meals web-based app, which was designed and developed by researchers according to the framework of the PECT-TurisTIC en Familia project;
(5) allergen content assessment, identified through the Healthy Meals web-based app;
(6) adequacy of vegetarian and vegan dishes, also evaluated through the Healthy Meals app.

As a secondary outcome, restaurant staff knowledge about the Mediterranean diet and food allergens was evaluated through paper-based questionnaires.

The primary outcomes were assessed as follows:

#### 2.3.1. AMed Criteria

The AMed criteria were designed by the Spanish Public Health Agency as the basis for an official certification that can be provided to restaurants and the establishment of a food service that guarantees the offering of a menu based on the Mediterranean diet [33]. The criteria are divided into nine mandatory and eight optional criteria (available at www.amed.cat, accessed on 20 January 2019) [33]. All 17 criteria were evaluated, but for the purpose of achieving the primary outcome, only the nine mandatory criteria were considered: (1) olive oil is used in dressings, and olive oil or high oleic sunflower are used for cooking; (2) 25% of the first course offerings are vegetables and/or legumes; (3) whole-grain products are included; (4) 50% of the second course offerings are based on fish, seafood or lean meat; (5) 50% of the dessert offerings are based on fresh fruit (whole or prepared); (6) dairy desserts without added sugar are offered; (7) free nonpackaged drinking water is offered; (8) wine, beer and cava are measured in glasses or individual units; (9) culinary preparations that do not require the addition of large amounts of fat, and culinary techniques that use little or no fat are used.

#### 2.3.2. Gluten Management (SMAP) Criteria

The SMAP criteria were developed by the Catalan Celiac Association [34] as the basis for obtaining the official SMAP certification for gluten-free food preparation. This recognition is intended to encourage restaurateurs to implement correct practices for allergen-free cooking and to avoid cross-contamination. The SMAP criteria include 18 recommendations that were evaluated in this study for assessing one of the primary outcomes [34].

#### 2.3.3. Nutritional Content Assessment of Restaurant-Offered Dishes

Dishes offered by the included restaurants were classified as starters, main dishes and desserts.

The nutritional quality of each meal was analysed through the Healthy Meals app using the recipes for each dish, including the ingredients used, their quantities and the cooking process. Home-spun ingredient measurements were converted to the equivalent quantity in grams. A food composition database for the extraction of the necessary nutritional data was generated from the nutritional information of commercial food products and data from different public databases [35,36,37,38,39]. The food ingredient database was used for the development of the Healthy Meals web-based app, which was used for the nutritional assessment of the restaurants’ dishes. The information obtained for each dish included the energy (Kcal), protein (g), total carbohydrates (g), sugar (g), total fat (g), saturated fat (g), fibre (g) and sodium (mg).

From the nutritional information calculated, a traffic light rating system for a single-plate portion was created; the system classified the content of each nutrient according to three colours, namely red (high), orange (medium) or green (good), in agreement with the cut-offs of the UK Food Standards Agency [40]. Based on a single-plate portion, nutrients were classified as (a) green when the dish contained <7.5% of the European Guideline Daily Amount (GDA); (b) orange when the dish contained between 7.5–20% of the GDA; (c) red when the dish contained >20% of the GDA recommended daily nutrient amounts for a healthy adult diet of 2000 Kcal [41]. However, the fibre content was classified inversely so that a red label corresponded to a low fibre content according to the recommendations.

Single dishes from the included restaurants were then evaluated through the traffic light system, and a number of healthy meals were identified as green-light dishes.

#### 2.3.4. Allergen Assessment

For each of the 297 dishes, the following 14 most common food allergens that should be declared according to European Regulation 1169/2011 [16] were identified taking into account the ingredients used and the cooking process: (1) cereals containing gluten, (2) milk, (3) eggs, (4) fish, (5) crustaceans, (6) tree nuts, (7) peanuts, (8) soya, (9) celery, (10) mustard, (11) sesame, (12) sulphites, (13) lupin and (14) molluscs.

#### 2.3.5. Adequacy for Vegetarian and Vegan Diet

According to the ingredients used, vegetarian- and vegan-adapted meals were identified. In particular, plant-based meals not containing animal products were labelled vegetarian, while meals not containing animal products or their derivates were marked vegan [42].

#### 2.3.6. Restaurant Staff Knowledge about the Mediterranean Diet and Food Allergens

Restaurant staff were divided into cooks and waiters, and their knowledge was evaluated according to two topics: Mediterranean diet and food allergens. The questionnaires used were adapted from AMed to evaluate Mediterranean diet knowledge [33] and from those designed by McAdams B. et al. to evaluate food allergy knowledge; however, these questionnaires have not been validated [43]. The information collected about the waiters’ knowledge on the Mediterranean diet included the identification of foods adhering to the Mediterranean diet (10 items), and regarding food allergens, the waiters had to identify the following: (1) the presence of food allergens in common traditional meals (14 items), (2) the critical points of food allergen management (13 items), and (3) food allergy and intolerance reactions (20 items). The information collected about the knowledge of kitchen staff about the Mediterranean diet included their identification of (1) foods adhering to the Mediterranean diet (10 items), (2) healthy food (8 items), and (3) the AMed criteria (8 items). On the other hand, regarding food allergens, the kitchen staff had to identify (1) food allergens in common traditional meals (14 items) and (2) critical points of food allergen management (11 items).

Staff knowledge was evaluated on a 10-point scale for the two evaluated themes (Mediterranean diet and food allergens) and for a total knowledge score.

### 2.4. Additional Data

The following information about the included restaurants’ general characteristics and offered meals were collected: (1) type of restaurant; (2) capacity; (3) location; (4) years in operation; (5) frequency of menu changes over a year; (6) restaurant administration (owner and his/her family, owner and recruited staff, or a recruited manager); (7) type of cuisine; (8) menu labelling; (9) availability of child/daily/weekend menu; (10) number of employees; (11) quality of the foods by purchases cooks and owners (fish, meat, fruit, vegetable, eggs, oil) according to the store and the type of products purchased; (12) type and management of the training provided to employees; (13) weaknesses, points to be improved and differences from other restaurants; (14) availability of healthy meals on the menu according to the owner; (15) staff knowledge about the restaurant’s menu offerings; (16) methods to avoid cross-contamination. Moreover, the following information about the restaurant’s owner and employees (waiters and cooks) was collected: (1) years of experience of the owner and (2) gender, education and age of the employees.

### 2.5. Statistical Analysis

Data are presented as the mean ± standard deviation (SD) for continuous variables and as percentages for categorical variables. Student’s *t*-tests for continuous variables were used to calculate the cooks’ and waiters’ knowledge. Moreover, the chi^2^ test was used for categorical variables to calculate the difference in green- vs. red-light dishes, among starters, main dishes and desserts. Bonferroni tests were conducted for differences among restaurant employees (owners, cooks and waiters) with their education degree and gender, and type of dishes (starters, main dishes and desserts) with gluten-free and allergen-free options.

The Pearson (r) correlation coefficient for variables with normal distribution and the Spearman (ρ) correlation coefficient for not normally distributed variables [44] were used to analyse the correlations between staff knowledge and restaurant compliance with the AMed and SMAP criteria and between the presence of green-light nutrients according to the traffic light system and AMed criteria. Statistical analysis was performed using SPSS software (version 26), and the significance level was fixed at *p* ≤ 0.05.

## 3. Results

**Figure 1:**
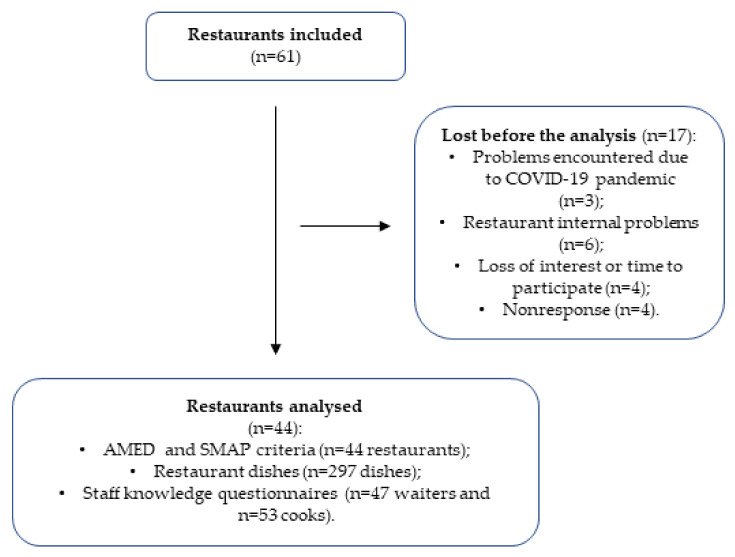
Study flow diagram according to the STROBE statement.

A total of 61 restaurants were recruited for the present cross-sectional analysis; however, 17 restaurants were excluded before the collection of the baseline data because of problems encountered due to the COVID-19 pandemic (*n* = 3), restaurant internal problems (*n* = 6), loss of interest or time to participate (*n* = 4), and nonresponse (*n* = 4) (Figure 1). As a result, 44 restaurants were analysed, as shown in Figure 1, and 297 dishes were analysed from 32 restaurants. A total of 47 questionnaires on staff Mediterranean diet and food allergen knowledge were collected from waiters, and 53 were collected from cooks.

### 3.1. General Characteristics of the Included Restaurants

Of the 44 included restaurants, the majority were urban (68.2%; *n* = 30) and coastal (18.2%; *n* = 8), offering dishes from both menus and daily specials (54.5%; *n* = 24). Restaurants had a medium average size of 134.1 ± 86.8 m^2^ with 17.7 ± 10.8 tables available to receive 61.7 ± 38.7 customers (Table 1). Twenty-two of the included restaurants were located in Tarragonès County which has the highest population density, including the cities of Tarragona, Salou and Torredembarra (50.0%); 17 were located in Baix Camp, which is the second most populated county [31], including the cities of Reus, Cambrils, Prades and Vinyols (38.6%); the others were located in different counties of the Tarragona Province with the least population density (Table 1). The restaurants had been open for 13.2 ± 18.9 years, offering the same menus throughout the entire year (25.0%; *n* = 11) or changing their offered dishes two or more times a year (68.1%; *n* = 30). Half of the included restaurants were run by the owner and his/her family (50.0%; *n* = 22) or by the owner with recruited staff (38.6%; *n* = 17), with an average of 5.4 ± 4.2 employees (Table 1).

The included restaurants reported offerings of Mediterranean cuisine (72.7%; *n* = 32), traditional cuisine (54.5%; *n* = 24), Catalan cuisine (43.2%; *n* = 19) and Spanish cuisine (36.4%; *n* = 16). In particular, 79.5% (*n* = 35 of 42 respondents) considered their menus to offer healthy meal options. None of the restaurants presented nutritional information, such as the traffic light labels, GDAs or healthier choice indicators, on the menu, while 29.5% (*n* = 13) identified the presence of allergens or gluten-free options, vegan/vegetarian options or traditional dishes prepared using local food. Only 29.5% (*n* = 13) offered children’s menus; on the other hand, 77.3% (*n* = 34) had a daily menu, and 56.8% (*n* = 25) had weekend menu offerings (Table 1). The majority of the included restaurants recognized that there were points that could be improved regarding the availability of allergen-adapted options (38.6%; *n* = 17 of 20 respondents) and menu offerings (29.6%; *n* = 13 of 17 respondents), and most did not see the lack of allergen-free options as a point of weakness or an area of competition with other restaurants (81.8%; *n* = 36 of 41 respondents). Meanwhile, the quality of restaurant service (72.7%; *n* = 32 of 41 respondents) and the quality of gastronomic offerings (63.6%; *n* = 28 of 41 respondents) were meant to be competitive differences with respect to other restaurants.

A significant positive correlation was observed between restaurateurs who considered their menu to be based on healthy offerings and the total AMed number of criteria fulfilled (ρ = 0.32; *p*-value = 0.04) [44]. Although the association was weak, when the restaurateur’s perception of the healthiness of the menu was positive, the number of AMed criteria fulfilled was higher.

### 3.2. General Characteristics of the Included Restaurant Owners and Their Employees

As shown in Table 2, the restaurant owners had an average of 18.0 ± 10.6 years of experience in the catering sector. Most of the restaurant owners were men (59.1%; *n* = 26), had primary (25.0%; *n* = 11) or secondary (52.3%; *n* = 23) education and were 44.0 ± 8.6 years old. Similarly, employees such as cooks and waiters were mainly men (67.9%, *n* = 36, and 53.2%, *n* = 25, respectively), had secondary education (60.4%, *n* = 32, and 59.6%, *n* = 28, respectively) and were 40.0 ± 11.4 and 38.3 ± 12.1 years old, respectively. However, differences in gender and education among owners, cooks and waiters were not statistically significant (*p* > 0.05) (Table 2).

### 3.3. AMed Criteria

As shown in Table 3, the 44 included restaurants had fulfilled an average (mean ± SD) of 5.1 ± 1.6 out of 9 AMed compulsory criteria. In particular, 93.2% (*n* = 41) had culinary preparations that did not require the addition of large amounts of fats, and 75.0% (*n* = 33) used olive oil in dressings and olive oil or high oleic sunflower oil for cooking. However, only 34.1% (*n* = 15) of the restaurants offered whole-grain products, and only 2.3% (*n* = 1) of the restaurants had 50% of dessert offerings based on fresh fruits.

Regarding the AMed optional criteria, the included restaurants fulfilled an average of 6.5 ± 0.9 out of 8 AMed criteria (Table 3). In particular, (a) all the restaurants (*n* = 44) prioritized fresh seasonal and local foods, (b) 95.5% (*n* = 42) offered traditional and local cuisine on their menus and (c) 93.2% (*n* = 41) offered virgin olive oil at tables. Consequently, the 44 included restaurants fulfilled an average of 11.5 ± 2.1 of the 17 total AMed compulsory and optional criteria (Table 3).

### 3.4. SMAP Criteria

The 44 included restaurants fulfilled an average of 12.9 ± 2.8 of the 18 total SMAP criteria (Table 4), demonstrating that there are still six pending criteria to improve for avoiding gluten cross-contamination. The most fulfilled criteria were related to the use of different tools or non-cross-contaminated kitchen tools for gluten food preparation (95.5%; *n* = 42), and to the attention of the staff in cleaning the kitchen area and their hands before the preparation of gluten-free food (97.7%; *n* = 43); however, only 38.6% (*n* = 17) had exclusive equipment (fryers, ovens, microwaves, etc.) for gluten-free food preparation, and only 45.5% (*n* = 20) used closed salt shakers and spice boxes which are recommended to avoid cross-contamination. Furthermore, very few restaurants (29.5%; *n* = 13) avoided the use of kitchen cloths and wooden tools, which are materials that can retain traces of gluten, or offered seasonings for exclusive use or in single-dose portions, that are very useful tools to preserve consumers’ safety (18.2%; *n* = 8) (Table 4).

### 3.5. Traffic Light and Nutritional Content Assessment of the Restaurants’ Offered Dishes

Nutritional content assessment was performed for 297 dishes, whose nutrient content was measured according to the three-colour traffic light system as an easy tool to assess the nutritional quality of dishes. A green-light evaluation good indicates nutritional quality in line with the GDAs recommendation for a single portion. Of the 297 assessed dishes, *n* = 119 were starters, *n* = 138 were main dishes and n = 40 were desserts. Based on the traffic light nutritional assessment, the 119 starter dishes were mainly rated green for carbohydrates (55.5%; *n* = 66), whereas few dishes were rated green for fibre (27.7%; *n* = 33), sodium (13.4%; *n* = 16), energy (10.1%; *n* = 12) or fat (5.9%; *n* = 7) (Table 5). Regarding the 138 main dishes, only 9.4% (*n* = 13) were rated green for saturated fat, 5.8% (*n* = 8) were rated green for sodium and 1.4% (*n* = 2) were rated green for total fat (Table 5). Regarding the 40 desserts, few were rated green for fibre (20.0%; *n* = 8) or sugar (2.5%; *n* = 1) (Table 5).

Comparing green- and red-light dishes (data not shown), no significant difference was observed for starters, main dishes and desserts (*p* > 0.05). Correlation analysis of the total number of AMed criteria fulfilled and green-light dishes showed a significant positive, weak correlation between AMed criteria and sugar (ρ = 0.32; *p*-value = 0.04), as well as a significant moderate correlation between AMed criteria and total fat (ρ = 0.57; *p*-value = 0.03), indicating that a greater number of AMed criteria are fulfilled when sugar and fat nutrients are rated green in the traffic light system (Table S2). However, a significant weak negative correlation was detected between AMed criteria and fibre (ρ = −0.32; *p*-value = 0.03). A greater number of fulfilled AMed criteria were correlated with a non-green traffic light rating for fibre. No other significant correlations were observed (*p* > 0.05) (Table S2).

As shown in Table 5, nutrient content was assessed for the type of plate. According to the recommended GDAs for a single portion [41], starters had high contents of energy, protein, fat, saturated fat and sodium; main dishes had high contents of energy, protein, fat, saturated fat and sodium; desserts had high contents of sugar, fat and saturated fat [40].

### 3.6. Allergen Content Assessment and Vegetarian and Vegan Dish Adequacy

The 14 most common allergens were identified by type of plate for the 297 analysed dishes. With respect to the allergen content (Table 6), few dishes were completely allergen-free (9.8%; *n* = 29), with the majority being starters (12.6%; *n* = 15) and main dishes (8.0%; *n* = 11) (*p* = 0.02). In particular, 142 of the total 297 analysed dishes (47.8%) were gluten-free, especially starters (53.8%; *n* = 64) and main dishes (44.2%; *n* = 61) (*p* < 0.001). Differences for type of plate (starter, main dishes and desserts) between allergen-free and gluten-free dishes were also statistically significant (*p* < 0.001).

According to the adequacy of vegetarian and vegan dishes (Table 6), 36.1% of the starters (*n* = 43) were suitable for vegetarians, and 19.3% were suitable for vegans (*n* = 23). On the other hand, only 2.2% of the main dishes (*n* = 3) were adapted for vegetarians, and 0.7% were adapted for vegans (*n* = 1). Finally, 85.0% of the desserts were appropriate for a vegetarian diet (*n* = 34), and 12.5% were appropriate for a vegan diet (*n* = 5). The main dishes are the type of dishes that contain fewer vegetarian options, compared among them, starters and desserts (*p* < 0.01). Moreover, main dishes contain fewer vegan options compared to starters (*p* < 0.01). Contrarily, the highest vegetarian options are present on desserts, and the highest vegan options on starters, compared to other dishes (*p* < 0.01). (Table 6).

### 3.7. Knowledge of Restaurant Staff about the Mediterranean Diet and Food Allergens

The restaurant waiters’ and cooks’ knowledge of the principles of the Mediterranean diet and food allergens was evaluated (Table 7). Regarding the Mediterranean diet, waiters received 7.9 ± 1.7 points and cooks received 6.9 ± 1.7 points of a total of 10 points, with a significant difference between the two groups (*p* = 0.003). On the other hand, regarding food allergen knowledge, waiters scored 5.8 ± 1.7 points and cooks scored 5.5 ± 1.5 points of a total of 10 points, with no significant difference (*p* = 0.36). Finally, general knowledge assessed out of a total of 10 points (5 points for the Mediterranean diet and 5 points for food allergens) amounted to 6.7 ± 1.5 points for waiters and 6.0 ± 1.7 points for cooks, with a significant difference between the two groups (*p* = 0.03) (Table 7).

Moreover, there was no significant correlation between the AMed criteria score and restaurant staff knowledge about the Mediterranean diet (cooks ρ = −0.05, *p*-value > 0.05; waiters ρ = −0.05, *p*-value > 0.05) or between the SMAP criteria score and restaurant staff knowledge about food allergens (cooks ρ = −0.18, *p*-value > 0.05; waiters ρ = −0.14, *p*-value > 0.05).

### 3.8. Additional Data

#### Restaurants’ Purchased Foods

Preferred stores for food shopping by cooks and restaurateurs were wholesalers for fish (78.8%; *n* = 52/66), meat (78.8%; *n* = 52/66), fruit (66.7%; *n* = 44/66) and vegetables (63.6%; *n* = 42/66) (Table S3). Furthermore, 36.4% of the restaurants bought farm eggs (*n* = 24/66), and 27.3% bought pasteurized eggs (*n* = 18/66). Regarding oil, most of the restaurants bought extra virgin olive oil (84.8%; *n* = 56/66) or sunflower oil (57.6%; *n* = 38/66), while 22.7% also used other types of oils (*n* = 15/66), and 22.7% used virgin olive oil (*n* = 15/66) (Table S3). Extra virgin olive oil was used mainly for raw seasonings and sauces (90.9%; *n* = 60/66), grilled and roasted foods (46.9%; *n* = 31/66) and candied foods (39.4%; *n* = 26/66), while sunflower oil was the most used for frying (39.2%; *n* = 26/66) (Table S3).

## 4. Discussion

The present cross-sectional study provides data about Mediterranean diet-adherent, healthy and allergen-free offerings by 44 full-service restaurants located in the province of Tarragona and 297 dishes. Regarding Mediterranean diet offerings, the included restaurants fulfilled an average of 5.1 ± 1.6 out of 9 compulsory AMed criteria, demonstrating that further efforts by restaurants are needed to ensure that their gastronomic offerings adhere to the Mediterranean diet recommendations [33]. Regarding the traffic light assessment, the dishes analysed were mainly rated green for sugar but not for energy or total fat content.

Unsurprisingly, other previous studies have demonstrated that restaurants should improve the healthiness of their meals, as observed in the present cross-sectional analysis. For instance, an observational study in 2017 found that US restaurant meals exceeded the American Heart Association’s (AHA) criteria, which indicate good nutritional content in terms of calories, total fat, saturated fat, cholesterol and sodium [45]. Similarly, another study reported that 25 of 32 restaurants analysed did not meet the criteria of the Nutrition Environment Measures Survey for Restaurants (NEMS-R); the study assessed the factors that contribute to increasing healthy food choices in restaurants, such as the availability of, promotion of and signage about healthy meals on the menu [46].

Although eating outside of the home is a pleasurable event for consumers, especially in Spain, where restaurants serve as a context for social and familiar interaction [47], eating pleasure should not be considered an enemy of health; in this sense, restaurants should offer healthier meals to customers without sacrificing taste [48] and should recognize the role of “food as wellbeing” [49]. Specifically, the present cross-sectional analysis showed that many restaurants do not include whole foods and desserts with no added sugar or based on fresh fruit on their menus. The importance of whole grain consumption is based on the recommended daily consumption of 2–3 servings per day of whole grains (±45 g/day) [50], as well as of fruit and vegetables (≥400 g/day) [51], which has been associated with a lower risk for developing noncommunicable diseases (NCDs), including cardiovascular diseases, diabetes type 2 and metabolic and gastrointestinal disorders [50,51]. However, the consumption of whole grains, fruit and vegetables worldwide still does not meet the recommended guidelines (38.4 g/day, 81.3 g/day and 208.8 g/day are actually consumed, respectively), thus constituting a global public health goal to be reached [52]. For instance, to help customers improve their daily consumption of whole grains and fresh fruits, restaurants should increase the availability of these healthy foods on their menus by directly substituting refined-grain foods with whole-grain foods [53] and serving fruit and vegetables as attractive side dishes or entrees [54]. Actually, a recent study about the changes that occurred in dietary habits of the Spanish population during the COVID-19 confinement revealed an increase in the consumption of Mediterranean diet-related foods such as olive oil, fruits, vegetables and legumes [55], reflecting that people desire to approach healthier dietary behaviours that restaurateurs should take into account. For instance, the closing of restaurant establishments could have improved people’s consumption, pointing out the lack of healthier offerings offered by restaurants.

According to the traffic light nutritional content assessment, the starters were mainly rated green for carbohydrates but did not meet the recommended GDAs for fibre, sodium, energy or total fat. These results confirm the observation regarding unfulfilled AMed criteria, pointing out that restaurants should provide more fruit, vegetables and whole-grain ingredients in first courses. Similarly, the main dishes were high in total fat, saturated fat and sodium, with only 59.1% of the restaurants providing fish, seafoods and lean meat as second courses; the latter strategy would result in lower fat content, as demonstrated by the significant correlation between fulfilment of the AMed criteria and green ratings for fat according to the traffic light system. Finally, few desserts were rated green for fibre and sugar since the restaurants did not include fresh fruits or pastries with no added sugars, as observed based on the unfulfilled AMed criteria. Furthermore, the average nutrient content was higher than the recommended GDA values per single portion. Similarly, a Canadian cross-sectional analysis found that sit-down restaurant meals were high in calories, saturated fat and sodium with respect to the recommended daily values and that these contents were even significantly higher than those of fast-food restaurant meals [20]. To decrease caloric content, an effective strategy could be substituting fat and caloric foods with greater portions of vegetables, which improves the healthiness and sustainability of the diet [56].

Furthermore, none of the included restaurants presented nutritional information on the menu, such as GDAs and healthy choice indicators or symbols, and the majority of the restaurateurs (79.5%) considered their menus already to offer healthy meals. Previous studies have demonstrated that there is a growing demand for nutritional labels on restaurant menus, especially by consumers between 35 and 65 years of age with healthy lifestyles who frequently eat outside of the home. In this sense, restaurants should be proactive and responsive to the request of an important part of the customer population through the design of menus with nutritional information to encourage healthier choices [57].

In relation to the food allergen content, the restaurants included in the present cross-sectional analysis offered different allergen-free options on their menus and considered the availability of appropriate meals for people with food allergies as a point to be improved, but they did not recognize the lack of allergen-free options as a weakness (81.8%). The prevalence of food allergies in Europe relates to cow’s milk, egg, wheat, soy, peanut, tree nuts, fish and shellfish [58]. In particular, although wheat is the main source of food in the world, providing up to 50% of the daily caloric intake in developed and developing countries [59], it is also the main source of gluten, whose disorders already affect the 1.4% of the global population [60]. For people who cannot consume foods containing gluten, the possibility to choose gluten-free meals when eating outside the home represents an important factor for their quality of life [61]; thus, restaurateurs should come across to these needs, improving menu offerings’ options and communication [61]. However, as observed from the present cross-sectional analysis, only 29.5% of the restaurants identified food allergens on their menus, and only 9.8% of the offered dishes were allergen-free, despite European Union legislation recommending the disclosure of the 14 most common allergens [16]. On the other hand, 47.8% of the offerings are gluten-free, pointing out that restaurants are starting to take more account of this emerging consumers’ need regarding food allergies.

The assessment of staff knowledge indicated that the waiters scored significantly higher than cooks for knowledge of the Mediterranean diet and total knowledge. On the other hand, concerning food allergen knowledge, both waiters and cooks received a barely passing score (5.8 ± 1.7 and 5.5 ± 1.5, respectively, out of 10 total points), and only 43.1% of the restaurateurs reported attending more than one training course on allergen management. Based on the described experience, although proper training on food safety is provided to restaurant staff, these courses are not always effective and have little impact on overall allergen management since theoretical courses are not combined with appropriate practical demonstrations [62]. However, it is essential to provide efficient training to restaurant staff about food allergy prevention and response, in case of adverse reactions [63], and about celiac disease and gluten-free diet management [64], as cooks still have many knowledge gaps in this area [65]. In the management of food allergens, the restaurants included in the present analysis fulfilled an average of 12.9 ± 2.8 SMAP criteria out of the 18 total recommendations defined by the Catalan Celiac Association [34], highlighting that restaurants were generally careful to avoid cross-contamination in the kitchen and dining room. However, few restaurants were equipped with tools for exclusive gluten-free preparations (ovens, fryers, etc.) or took special precautions such as providing single-serving dressings for allergic customers and closed containers for salt and spices. Although different studies have demonstrated that shared ovens for the cooking of gluten-free and gluten-containing pizza [66], as well as of kitchen utensils as spoons and knives [67], do not pose a relevant risk when specific requirements are complied, these tools could undergo cumulative contamination throughout the day and involve a higher risk during dinner service [68]. Thus, further precautions should be taken by restaurants for the preparation of allergen-free food to avoid adverse reactions and the endangerment of consumers’ lives [69].

Additionally, few restaurants indicated meals suitable for vegetarian and vegan diets (29.5%) on their menus, and the offerings were very limited (26.9 and 9.8%, respectively). Although restaurants still have limited plant-based meal options, in 2019, 1.5% and 0.5% of the Spanish population reported following a vegetarian or a vegan diet, respectively [70], while in 2020, the sales volume of vegetarian and vegan food products in Spanish supermarkets increased by 20% [71]. Thus, as demonstrated by a recent study, the increase in vegetarian and vegan meal options could have a positive impact both on meal sales and on the improvement of consumers’ sustainable food choices and satisfaction [72].

Finally, few of the included restaurants in this cross-sectional analysis offered child menus (29.5%), but the majority of them would prepare half-portion meals (88.6%). However, as suggested by previous studies, regarding the offering of half portions or smaller serving sizes of adult dishes to children, restaurants should include healthier options to accustom children to selecting and consuming healthy meals [73,74]. Similarly, as reported by Mueller et al., the improvement of healthy child menus seems to encourage the selection of healthier meals by adults [75].

Based on the present cross-sectional analysis, several recommendations could be proposed to restaurants to increase Mediterranean menu offerings and improve food allergen management (Table S4). In particular, when limited resources can be invested in the development of new offerings, as is the case for most independent restaurants that operate with narrower profit margins [13], restaurants should improve the promotion of existing healthy options and encourage consumers to select such offerings through symbols and indicators on menus.

Similarly, regarding food allergen management, according to the present analysis, restaurants should provide more training courses to their staff and provide more information to customers about the presence of food allergens on the menu (Table S4). On the other hand, several positive practices were observed at the included restaurants: the use of extra virgin olive oil for dressing and cooking, which has been associated with many health positive effects due to the high content of oleic acid and bioactive compounds (e.g., polyphenols) [76], as well as the preference of fresh seasonable and traditional fruit and vegetables, which contribute to a sustainable diet [77,78] (Table S4).

Several notable limitations were encountered in the present cross-sectional analysis. First, there were difficulties experienced during data collection since many restaurants did not respond or withdrew from the study before the beginning of data collection. Second, the present cross-sectional study occurred in the midst of the SARS-CoV-2 pandemic, limiting the participation and inclusion of restaurants in the study, many of which closed during the pandemic. Third, due to the pandemic, the restaurant industry has adapted its offerings according to national dispositions, so the current data could differ from the presented analysis. Fourth, since the majority of the included restaurants in the present study were urban, the inclusion of more rural and coastal restaurants in the same proportion would homogenize the results more, making them more representative of the province of Tarragona. Fifth, data collection methods are validated in our local area, being designed and developed by Catalan entities for local realities. However, the use of these data collection methods limited the comparison among international studies. Finally, some restaurant information was lost due restaurants not completing the questionnaires or not providing all the information requested.

## 5. Conclusions

In conclusion, the restaurants did not meet all of the AMed and SMAP criteria. An increase in fibre and a decrease in saturated fat content are needed to improve the nutritional quality of dishes and the consumers’ adherence to healthy diets. Additionally, for restaurant staff, training courses on food allergens should be considered to improve the allergen management in restaurants. Furthermore, more information should be provided to customers on menus about the presence of food allergens, healthier food choices and vegetarian and vegan options.

## Figures and Tables

**Table 1:** General characteristics of the included restaurants.

	*N* = 44% (*n*)
Restaurant type:	
Rural	13.6 (6)
Urban	68.2 (30)
Coastal	18.2 (8)
Restaurant offer:	
Daily menu	22.7 (10)
Menu	22.7 (10)
Daily menu + menu	54.5 (24)
Location:	
Tarragonès	50.0 (22)
Baix Camp	38.6 (17)
Montsià	7.0 (3)
Baix Penedès	2.3 (1)
Conca de Barberà	2.3 (1)
Time of restaurant activity in years ^2^:	13.2 ± 18.9
Frequency of menu changes:	
Twice a year (winter/summer)	22.7 (10)
More than twice a year	22.7 (10)
It is the same throughout the year	25.0 (11)
Other	22.7 (10)
Not answered	6.8 (3)
Administration of the restaurant:	
The owner with his/her family	50.0 (22)
The owner with recruited staff	38.6 (17)
Recruited manager	4.5 (2)
The owner with his family and the recruited staff	4.5 (2)
Not answered	2.3 (1)
Number of recruited employees ^2^:	5.4 ± 4.2
Type of cuisine ^1^:	
Traditional	54.5 (24)
Spanish	36.4 (16)
Catalan	43.2 (19)
Mediterranean	72.7 (32)
Author	18.2 (8)
Italian	13.6 (6)
Fusion	15.9 (7)
Tapas	31.8 (14)
Other	11.4 (5)
Presence on the menu of ^1^:	
Nutritional information	0.0 (0)
Traffic light labels	0.0 (0)
GDAs	0.0 (0)
Healthier choice indicators	0.0 (0)
Colours	6.8 (3)
Other (allergens, vegan/vegetarian/celiac options, typical cuisine meals, prepared with local products)	29.5 (13)
Availability of a children’s menu:	
Yes	29.5 (13)
No	70.5 (31)
Availability of daily menu:	
Yes	77.3 (34)
No	22.7 (10)
Availability of a weekend menu:	
Yes	56.8 (25)
No	43.2 (19)

^1^: Total percentage of respondents is higher than 100% due to the multiple-option responses given by the restaurateurs and cooks; ^2^: data are expressed in Mean ± SD.

**Table 2:** General characteristics of the included restaurant owners and employees.

	Total ^1^ (*N* = 144) % (*n*)	Owners ^2^ (*N* = 44) % (*n*)	Cooks ^2^ (*N* = 53) % (*n*)	Waiters ^2^ (*N* = 47) % (*n*)
Gender:				
Men	60.4 (87)	59.1 (26)	67.9 (36)	53.2 (25)
Women	28.5 (41)	18.2 (8)	28.3 (15)	38.3 (18)
Not answered	11.1 (16)	22.7 (10)	3.8 (2)	8.5 (4)
Education degree:				
Uneducated	1.4 (2)	0.0 (0)	1.9 (1)	2.1 (1)
First grade studies	21.5 (31)	25.0 (11)	18.9 (10)	21.3 (10)
Second grade studies	57.6 (83)	52.3 (23)	60.4 (32)	59.6 (28)
Third grade studies	16.7 (24)	18.2 (8)	18.9 (10)	12.8 (6)
Not answered	2.8 (4)	4.5 (2)	0.0 (0)	4.3 (2)
Experience of the restaurant owners in the catering sector ^3^:				
Years		18.0 ± 10.6		

^1^: Total population is referred to the sum of restaurant owners (*n* = 44), cooks and kitchen staff (*n* = 53) and waiters (*n* = 47); ^2^: Bonferroni tests were conducted for differences among restaurant owners, cooks and waiters (significance: *p* < 0.05); ^3^: Data are expressed in Mean ± SD.

**Table 3:** Compulsory and optional AMed criteria fulfilled by the included restaurants.

	*N* = 44 % (*n*)
AMed Compulsory criteria:	
(1) olive oil for dressing, and olive oil or high oleic sunflower for cooking	75.0 (33)
(2) 25% of the first course offerings are vegetables and/or legumes	70.5 (31)
(3) presence of whole-grain products	34.1 (15)
(4) 50% of the second course offerings based on fish, seafood and lean meat	59.1 (26)
(5) 50% of the dessert offerings based on fresh fruit (whole or prepared)	2.3 (1)
(6) offer of dairy desserts without added sugar	43.2 (19)
(7) offer of free nonpackaged drinking water	31.8 (14)
(8) wine, beer and cava are measured in glasses or individual units	100 (44)
(9) have culinary preparations that do not require the addition of large amounts of fat, and culinary techniques that use little or no fat are used	93.2 (41)
Number of total AMed compulsory criteria fulfilled per restaurant (mean ± SD) ^1^:	5.1 ± 1.6
AMed Optional criteria:	
(1) prioritize fresh seasonal and local foods	100 (44)
(2) include proposals of the traditional and local cuisine	95.5 (42)
(3) offer virgin olive oil at tables	93.2 (41)
(4) prioritize side dishes of vegetables and legumes	84.1 (37)
(5) offer the most symbolic recipes of the restaurant that accomplish the AMed criteria to their customers	11.4 (5)
(6) offer the option of unique dishes or medium portions	79.5 (35)
(7) offer options with no added salt	88.6 (39)
(8) disseminate information about leisure activities in the nearby environment	95.5 (42)
Number of total optional criteria fulfilled per restaurant (mean ±SD) ^2^:	6.5 ± 0.9
Number of 17 total AMed criteria fulfilled per restaurant (mean ±SD) ^3^:	11.5 ± 2.1

SD: Standard Deviation; AMed: Mediterranean Diet offer. ^1^: 9 compulsory AMed criteria; ^2^: 8 optional AMed criteria; ^3^: 17 total AMed criteria.

**Table 4:** SMAP criteria fulfilled by the included restaurants.

	*N* = 44 %(*n*)
(1) working with suppliers who guarantee their products do not contain gluten, on labels or data sheets	75.0 (33)
(2) store gluten-free products separately from products containing gluten in closets, freezers or refrigerators	77.3 (34)
(3) transfer to hermetically closed containers after opening, and identify the content	86.4 (38)
(4) keep flours and breadcrumbs containing gluten tightly closed and store them away from other food products	70.5 (31)
(5) use different or non-cross-contaminated kitchen tools for gluten-free food preparation	95.5 (42)
(6) be provided of equipment for the exclusive use of gluten-free food preparation (fryers, ovens, microwaves, toasters, sandwich makers, pasta makers)	38.6 (17)
(7) preparation of gluten-free plates before the other food preparation	81.8 (36)
(8) have a differentiated area within the kitchen for the preparation of gluten-free food, or a good working protocol	68.2 (30)
(9) cleaning of the food preparation area before starting to work	97.7 (43)
(10) cleaning of the hands before starting the preparation of all the necessary ingredients	97.7 (43)
(11) be provided of a clean apron, if you work with flours or products that may leave gluten traces on the clothes	54.5 (24)
(12) dispose of closed salt shakers and spice boxes, or use a teaspoon to pick up the salt as long as hands will be not placed inside	45.5 (20)
(13) do not use kitchen cloths and wooden tools, which are materials that can retain traces of gluten	29.5 (13)
(14) do not reuse oil, cooking water or broths when gluten products have been cooked, and do not share spreads foods	86.4 (38)
(15) serve gluten-free food in dishes of different colours or easy to identify (e.g., flags), and cover up until the moment of service	72.7 (32)
(16) re-prepare gluten-free dishes if a potential contamination occurs with a gluten-containing product	95.5 (42)
(17) place on the tables bottles of oil and vinegar, sauces and baskets for exclusive use, or single-dose portions	18.2 (8)
(18) cleaning of the waiter’s hands before the serving of the gluten-free dishes, to be taken	97.7 (43)
Number of total criteria fulfilled per restaurant (mean ± SD) ^1^:	12.9 ± 2.8

^1^: 18 SMAP criteria in total.

**Table 5:** Traffic light and nutritional content assessment of the restaurants’ offered dishes.

**Number of Green-Light Dishes per Nutrient and Type of Plate ^1^:**				
	**Total** **(*n* = 297)** **% (*n*)**	**Starters** **(*n* = 119)** **% (*n*)**	**Main dishes** **(*n* = 138)** **% (*n*)**	**Desserts** **(*n* = 40)** **% (*n*)**
Energy	7.7 (23)	10.1 (12)	3.6 (5)	15.0 (6)
Carbohydrates	47.1 (140)	55.5 (66)	45.7 (63)	27.5 (11)
Sugar	59.9 (178)	63.0 (75)	73.9 (102)	2.5 (1)
Protein	10.1 (30)	14.3 (17)	1.4 (2)	27.5 (11)
Total fat	6.1 (18)	5.9 (7)	1.4 (2)	22.5 (9)
Saturated fat	14.8 (44)	19.3 (23)	9.4 (13)	20.0 (8)
Sodium	18.2 (54)	13.4 (16)	5.8 (8)	75.0 (30)
Fibre	25.9 (77)	27.7 (33)	26.1 (36)	20.0 (8)
**Nutrient content of the restaurant dishes ^2^:**				
	**Total** **(*n* = 297)** **Mean ± SD**	**Starters** **(*n* = 119)** **Mean ± SD**	**Main dishes** **(*n* = 138)** **Mean ± SD**	**Desserts** **(*n* = 40)** **Mean ± SD**
Energy (Kcal)	490.3 ± 310.2	442.5 ± 290.8	566.3 ± 319.3	368.9 ± 271.4
Carbohydrates (g)	27.7 ± 26.5	26.6 ± 28.7	25.5 ± 23.8	38.7 ± 26.4
Sugar (g)	10.0 ± 13.2	6.8 ± 6.8	6.6 ± 7.4	31.3 ± 21.4
Protein (g)	25.5 ± 21.1	19.7 ± 17.3	35.5 ± 21.5	8.5 ± 10.1
Total fat (g)	30.2 ± 24.3	27.5 ± 21.6	35.7 ± 26.8	19.4 ± 17.6
Saturated fat (g)	8.5 ± 8.3	7.7 ± 8.8	8.8 ± 7.6	10.2 ± 9.4
Sodium (mg)	950.4 ± 814.4	1030.5 ± 836.8	1107.7 ± 778.0	169.6 ± 237.9
Fibre (g)	3.8 ± 4.0	4.0 ± 3.5	4.0 ± 4.4	2.2 ± 3.1

^1^: % (*n*); ^2^: Data are expressed in Mean ± SD, and values are referred to a single portion.

**Table 6:** Allergen content assessment and vegetarian and vegan dish adequacy.

	Total ^1^ (*n* = 297) % (*n*)	Starters ^1,2^ (*n* = 119) % (*n*)	Main dishes ^1,2^ (*n* = 138) % (*n*)	Desserts ^1,2^ (*n* = 40) % (*n*)
Allergen-free ^3^	9.8 (29)	12.6 (15)	8.0 (11)	7.5 (3)
Gluten-free ^3^	47.8 (142)	53.8 (64)	44.2 (61)	42.5 (17)
Vegetarian ^4^	26.9 (80)	36.1 (43) *	2.2 (3) *	85.0 (34) *
Vegan ^4^	9.8 (29)	19.3 (23) *	0.7 (1) *	12.5 (5)

^1^: % (*n*); ^2^: Bonferroni tests were conducted for differences among type of dishes: starters, main dishes and desserts; ^3^: data refer to the dishes not containing the food allergen; ^4^: data refer to vegetarian and vegan-adapted dishes; *: *p*-value ≤ 0.05.

**Table 7:** Knowledge of restaurant staff about the Mediterranean diet and food allergens.

Mediterranean Diet Knowledge ^1^:	Total Staff ^2^ (Mean ± SD)	Waiters (Mean ± SD)	Cooks (Mean ± SD)	*p*-Value ^3^
1. Identification of foods adhering to the Mediterranean diet		7.9 ± 1.7	8.0 ± 1.4	0.94
2. Identification of healthy food			6.8 ± 1.2	
3. Identification of the AMed compulsory criteria			7.7 ± 1.7	
Total score about Mediterranean diet ^1^	7.4 ± 1.8	7.9 ± 1.7	6.9 ± 1.7	0.003 *
Food allergens knowledge ^1^:				
1. Identification of food allergens in traditional meals		3.2 ± 1.8		
2. Identification of the critical points of food allergen management		8.2 ± 1.7	3.6 ± 1.3	0.29
3. Identification of food allergy and intolerance reactions		8.0 ± 1.5	8.4 ± 1.4	0.55
Total score about food allergens ^1^	5.6 ± 1.6	5.8 ± 1.7	5.5 ± 1.5	0.36
Total score of staff knowledge ^1^	6.3 ± 1.7	6.7 ± 1.5	6.0 ± 1.7	0.03 *

AMed: Mediterranean Diet offer; SD: Standard Deviation. ^1^: Scores are calculated on a total of a maximum of 10 points; ^2^: Total staff includes restaurant waiters and cooks; ^3^: *t*-test; *: *p*-value ≤ 0.05.

## Data Availability

The data presented in this study are available on request from the corresponding author. The data are not publicly available due to restrictions of privacy and ethical.

## Supplementary Materials

The following are available online at https://www.mdpi.com/article/10.3390/nu13072464/s1. Table S1: STROBE checklist for observational cross-sectional studies; Table S2: Correlation analysis between total number of AMed criteria fulfilled and green-light dishes; Table S3: Restaurants’ purchased foods; Table S4: Recommendations for restaurants to increase Mediterranean menu offerings and improve food allergen management.

## Abbreviations

RCT: Randomized Controlled Trial; GDA: Guideline Daily Amounts; AMed: Mediterranean Diet; SD: Standard Deviation.

## References

[1] Adams J., Goffe L., Brown T., Lake A.A., Summerbell C., White M., Wrieden W., Adamson A.J. (2015). Frequency and socio-demographic correlates of eating meals out and take-away meals at home: Cross-sectional analysis of the UK national diet and nutrition survey, waves 1–4 (2008-12). Int. J. Behav. Nutr. Phys. Act..

[2] https://empresa.dinamig.cat/media/sites/2/2018/07/Estudi-de-la-Restauraci%C3%B3-a-Catalunya.-H%C3%A0bits-de-comportament-i-tend%C3%A8ncies-pdf.

[3] Orfanos P., Naska A., Rodrigues S., Lopes C., Freisling H., Rohrmann S., Sieri S., Elmadfa I., Lachat C., Gedrich K. (2017). Eating at restaurants, at work or at home. Is there a difference: A study among adults of 11 European countries in the context of the HECTOR∗ project. Eur. J. Clin. Nutr..

[4] Obbagy J.E., Essery E.V., USDA (2012). The Food Environment, Eating Out, and Body Weight: A Review of the Evidence. Nutr. Insight.

[5] Myhre J.B., Loken E.B., Wandel M., Andersen L.F. (2014). Eating location is associated with the nutritional quality of the diet in Norwegian adults. Public Health Nutr..

[6] Bes-Rastrollo M., Basterra-Gortari F.J., Snchez-Villegas A., Marti A., Martnez J.A., Martnez-González M.A. (2010). A prospective study of eating away-from-home meals and weight gain in a Mediterranean population: The SUN (Seguimiento Universidad de Navarra) cohort. Public Health Nutr..

[7] Choi M.K., Kim T.Y., Yoon J.S. (2011). Does frequent eating out cause undesirable food choices? Association of food away from home with food consumption frequencies and obesity among Korean housewives. Ecol. Food Nutr..

[8] Darmon N., Briend A., Drewnowski A. (2004). Energy-dense diets are associated with lower diet costs: A community study of French adults. Public Health Nutr..

[9] Creel J.S., Sharkey J.R., McIntosh A., Anding J., Huber J.C. (2008). Availability of healthier options in traditional and nontraditional rural fast-food outlets. BMC Public Health.

[10] An R. (2013). Effectiveness of subsidies in promoting healthy food purchases and consumption: A review of field experiments. Public Health Nutr..

[11] Hwang J., Lorenzen C.L. (2008). Effective nutrition labeling of restaurant menu and pricing of healthy menu. J. Foodserv..

[12] Nothwehr F., Snetselaar L., Dawson J.D., Hradek C., Sepulveda M. (2010). Healthy Option Preferences of Rural Restaurant Customers. Health Promot. Pract..

[13] Fuster M., Handley M.A., Alam T., Fullington L.A., Elbel B., Ray K., Huang T.T.K. (2021). Facilitating healthier eating at restaurants: A multidisciplinary scoping review comparing strategies, barriers, motivators, and outcomes by restaurant type and initiator. Int. J. Environ. Res. Public Health.

[14] Versluis A., Knulst A.C., Kruizinga A.G., Michelsen A., Houben G.F., Baumert J.L., van Os-Medendorp H. (2015). Frequency, severity and causes of unexpected allergic reactions to food: A systematic literature review. Clin. Exp. Allergy.

[15] Banerjee D.K., Kagan R.S., Turnbull E., Joseph L., St. Pierre Y., Dufresne C., Gray-Donald K., Clarke A.E. (2006). Lunch Guidelines Are Effective in Reducing Peanut Substances in Primary School Classrooms in Montreal, Canada. J. Allergy Clin. Immunol..

[16] (2011). The European Parliment and the Council of the European Union Regulation (EU) No 1169/2011 on the provision of food information to consumers. Off. J. Eur. Union.

[17] Begen F.M., Barnett J., Payne R., Gowland M.H., DunnGalvin A., Lucas J.S. (2018). Eating out with a food allergy in the UK: Change in the eating out practices of consumers with food allergy following introduction of allergen information legislation. Clin. Exp. Allergy.

[18] Urban L.E., Lichtenstein A.H., Gary C.E., Fierstein J.L., Equi A., Kussmaul C., Dallal G.E., Roberts S.B. (2013). The energy content of restaurant foods without stated calorie information. JAMA Intern. Med..

[19] Theis D.R.Z., Adams J. (2019). Differences in energy and nutritional content of menu items served by popular UK chain restaurants with versus without voluntary menu labelling: A cross-sectional study. PLoS ONE.

[20] Murphy S.A., Weippert M.V., Dickinson K.M., Scourboutakos M.J., L’Abbé M.R. (2020). Cross-Sectional Analysis of Calories and Nutrients of Concern in Canadian Chain Restaurant Menu Items in 2016. Am. J. Prev. Med..

[21] Tatli M., Akoğlu A. (2020). Food Allergy Knowledge, Attitude and Practices of Restaurant Employees in Istanbul, Turkey. Sidas Medya.

[22] Loerbroks A., Tolksdorf S.J., Wagenmann M., Smith H. (2019). Food allergy knowledge, attitudes and their determinants among restaurant staff: A cross-sectional study. PLoS ONE.

[23] Bailey S., Albardiaz R., Frew A.J., Smith H. (2011). Restaurant staff’s knowledge of anaphylaxis and dietary care of people with allergies. Clin. Exp. Allergy.

[24] Pardo J.E., Sena E., Cuesta M.A., Granell J.D., Valiente J., Alvarez-Ortí M. (2013). Evaluation of potential and real quality of virgin olive oil from “campos de Hellín” (Albacete, Spain). J. Am. Oil Chem. Soc..

[25] De la Torre-Robles A., Rivas A., Lorenzo-Tovar M.L., Monteagudo C., Mariscal-Arcas M., Olea-Serrano F. (2014). Estimation of the intake of phenol compounds from virgin olive oil of a population from southern Spain. Food Addit. Contam. Part A Chem. Anal. Control. Expo. Risk Assess..

[26] Buckland G., Mayén A.L., Agudo A., Travier N., Navarro C., Huerta J.M., Chirlaque M.D., Barricarte A., Ardanaz E., Moreno-Iribas C. (2012). Olive oil intake and mortality within the Spanish population (EPIC-Spain). Am. J. Clin. Nutr..

[27] Gaforio J.J., Visioli F., Alarcón-De-la-lastra C., Castañer O., Delgado-Rodríguez M., Fitó M., Hernández A.F., Huertas J.R., Martínez-González M.A., Menendez J.A. (2019). Virgin olive oil and health: Summary of the iii international conference on virgin olive oil and health consensus report, JAEN (Spain) 2018. Nutrients.

[28] World Medical Association Declaration of Helsinki-Medical Research Involving Human Subjects. https://www.wma.net/what-we-do/medical-ethics/declaration-of-helsinki/.

[29] U.S. National Library of Medicine ClinicalTrials.gov. https://clinicaltrials.gov/ct2/about-site/background.

[30] Von Elm E., Altman D.G., Egger M., Pocock S.J., Gøtzsche P.C., Vandenbroucke J.P. (2008). The Strengthening the Reporting of Observational Studies in Epidemiology (STROBE) statement: Guidelines for reporting observational studies. J. Clin. Epidemiol..

[31] Gencat-Instituto de Estadística de Cataluña Población Empadronada (2020). Por Tamaño del Municipio. Comarcas, ámbitos y Provincias. Population Registered. By Size of Municipality. Districts, Areas and Provinces. https://www.idescat.cat/pub/?id=aec&n=248&lang=es.

[32] Tripadvisor. https://www.tripadvisor.es/.

[33] Agència de Salut Pública de Catalunya (2007). Alimentación Mediterrània. Mediterranean Diet.

[34] Associació Celíacs de Catalunya SMAP Criteria Gluten Management. https://www.celiacscatalunya.org/es/index.php.

[35] RedBedca AESAN Base de Datos Española de Composición de Alimentos-BEDCA. Spanish Database of Food Composition-BEDCA. https://www.bedca.net/.

[36] Mcgraw-Hill (2003). Tablas De Composicion De Alimentos Del Cesnid. Cesnid Food Composition Tables.

[37] Verdú J.M. (2003). Tabla De Composición De Alimentos. Food Composition Table.

[38] Favier J.C., Ireland-Ripert J., Toque C., Feinberg M. (1995). Répertoire général des aliments: Table de composition. General Food Directory: Composition Table.

[39] Moreiras O., Carbajal Á., Cabrera L., Cuadrado C. (2013). Tablas de composición de alimentos. Food Composition Tables.

[40] Food Standards Agency, Department of Health, Scotland, Northern Ireland and Wales Governments (2016). Guide to Creating a Front of Pack (FoP) Nutrition Label for Pre-Packed Products Sold through Retail Outlets.

[41] Federación Española de Industrias de Alimentación y Bebidas (FIAB), Confederación Europea de Industrias de Alimentación y Bebidas (CIAA) (2008). Recomendación CIAA para un esquema común de etiquetado nutricional. CIAA Recommendation for a Common Nutrition Labeling Scheme.

[42] Tuso P.J., Ismail M.H., Ha B.P., Bartolotto C. (2013). Nutritional update for physicians: Plant-based diets. Perm. J..

[43] McAdams B., Deng A., MacLaurin T. (2018). Food allergy knowledge, attitudes, and resources of restaurant employees. Br. Food J..

[44] Schober P., Schwarte L.A. (2018). Correlation coefficients: Appropriate use and interpretation. Anesth. Analg..

[45] Alexander E., Rutkow L., Gudzune K.A., Cohen J.E., McGinty E.E. (2020). Healthiness of US Chain Restaurant Meals in 2017. J. Acad. Nutr. Diet..

[46] Lindberg R., Sidebottom A.C., McCool B., Pereira R.F., Sillah A., Boucher J.L. (2018). Changing the restaurant food environment to improve cardiovascular health in a rural community: Implementation and evaluation of the Heart of New Ulm restaurant programme. Public Health Nutr..

[47] Diaz-Mendez C., Garcia-Espejo I. (2017). Eating out in Spain: Motivations, sociability and consumer contexts. Appetite.

[48] Landry M., Lemieux S., Lapointe A., Bédard A., Bélanger-Gravel A., Bégin C., Provencher V., Desroches S. (2018). Is eating pleasure compatible with healthy eating? A qualitative study on Quebecers’ perceptions. Appetite.

[49] Cornil Y., Chandon P. (2016). Pleasure as an ally of healthy eating? Contrasting visceral and Epicurean eating pleasure and their association with portion size preferences and wellbeing. Appetite.

[50] McRae M.P. (2017). Health Benefits of Dietary Whole Grains: An Umbrella Review of Meta-analyses. J. Chiropr. Med..

[51] FAO/WHO (2004). Fruit and Vegetables for Health-Report of a Joint FAO/WHO Workshop.

[52] Micha R., Khatibzadeh S., Shi P., Andrews K.G., Engell R.E., Mozaffarian D. (2015). Global, regional and national consumption of major food groups in 1990 and 2010: A systematic analysis including 266 country-specific nutrition surveys worldwide. BMJ Open.

[53] Tritt A., Reicks M., Marquart L. (2015). Reformulation of pizza crust in restaurants may increase whole-grain intake among children. Public Health Nutr..

[54] Anzman-Frasca S., Dawes F., Sliwa S., Dolan P.R., Nelson M.E., Washburn K., Economos C.D. (2014). Healthier side dishes at restaurants: An analysis of children’s perspectives, menu content, and energy impacts. Int. J. Behav. Nutr. Phys. Act..

[55] Rodríguez-Pérez C., Molina-Montes E., Verardo V., Artacho R., García-Villanova B., Guerra-Hernández E.J., Ruíz-López M.D. (2020). Changes in dietary behaviours during the COVID-19 outbreak confinement in the Spanish COVIDiet study. Nutrients.

[56] Reinders M.J., Huitink M., Dijkstra S.C., Maaskant A.J., Heijnen J. (2017). Menu-engineering in restaurants—adapting portion sizes on plates to enhance vegetable consumption: A real-life experiment. Int. J. Behav. Nutr. Phys. Act..

[57] Josiam B., Foster C. (2009). Nutritional information on restaurant menus: Who cares and why restauranteurs should bother. Int. J. Contemp. Hosp. Manag..

[58] Nwaru B.I., Hickstein L., Panesar S.S., Roberts G., Muraro A., Sheikh A. (2014). Prevalence of common food allergies in Europe: A systematic review and meta-analysis. Allergy Eur. J. Allergy Clin. Immunol..

[59] Tovoli F., Masi C., Guidetti E., Negrini G., Paterini P., Bolondi L. (2015). Clinical and diagnostic aspects of gluten related disorders. World J. Clin. Cases.

[60] Singh P., Arora A., Strand T.A., Leffler D.A., Catassi C., Green P.H., Kelly C.P., Ahuja V., Makharia G.K. (2018). Global Prevalence of Celiac Disease: Systematic Review and Meta-analysis. Clin. Gastroenterol. Hepatol..

[61] Šálková D., Hes A. (2015). Gluten-free food—The influence of selected qualitative characteristics on consumer decision making of coeliacs in hospitality establishments. Czech. J. Food Sci..

[62] Roberts K.R., Barrett B.B., Howells A.D., Shanklin C.W., Pilling V.K., Brannon L.A. (2018). Food Safety Training and Foodservice Employees’ Knowledge and Behavior. Food Safey Train. Foodserv. Empl..

[63] van Dam M., Wiersma L. (2013). To what extent are restaurants prepared to respond to the needs of guests with food allergies and intolerances?. Res. Hosp. Manag..

[64] Young I., Thaivalappil A. (2018). A systematic review and meta-regression of the knowledge, practices, and training of restaurant and food service personnel toward food allergies and Celiac disease. PLoS ONE.

[65] Schultz M., Shin S., Coppell K.J. (2017). Awareness of coeliac disease among chefs and cooks depends on the level and place of training. Asia Pac. J. Clin. Nutr..

[66] Vincentini O., Izzo M., Maialetti F., Gonnelli E., Neuhold S., Silano M. (2016). Risk of Cross-Contact for Gluten-Free Pizzas in Shared-Production Restaurants in Relation to Oven Cooking Procedures. J. Food Prot..

[67] Studerus D., Hampe E.I.L.G., Fahrer D., Wilhelmi M., Vavricka S.R. (2018). Cross-contamination with gluten by using kitchen utensils: Fact or fiction?. J. Food Prot..

[68] Lerner B.A., Vo L.P., Yates S., Rundle A.G., Lebwohl B., Green P.H.R. (2019). Detection of Gluten in Gluten-Free Labeled Restaurant Food: Analysis of Crowd-Sourced Data. Am. J. Gastroenterol..

[69] Pádua I., Moreira A., Moreira P., Barros R. (2016). Food allergy: Practical approach on education and accidental exposure prevention. Eur. Ann. Allergy Clin. Immunol..

[70] Medina A. (2019). Spain Spanish Consumers Grow Interest in Free From Functional Foods.

[71] Smart Protein Project (2021). Plant.-Based Foods in Europe: How Big Is the Market?. The Smart Protein Plant.

[72] Garnett E.E., Balmford A., Sandbrook C., Pilling M.A., Marteau T.M. (2019). Impact of increasing vegetarian availability on meal selection and sales in cafeterias. Proc. Natl. Acad. Sci. USA.

[73] Mcguf L.E., Price R.K., Mccaffrey T.A., Hall G., Lobo A., Wallace J.M.W., Livingstone M.B.E. (2014). Parent and child perspectives on family out-of-home eating: A qualitative analysis. Public Health Nutr..

[74] Anzman-Frasca S., Folta S.C., Glenn M.E., Jones-Mueller A., Lynskey V.M., Patel A.A., Tse L.L., Lopez N.V. (2017). Healthier Children’s Meals in Restaurants: An Exploratory Study to Inform Approaches That Are Acceptable Across Stakeholders. J. Nutr. Educ. Behav..

[75] Mueller M.P., Shonkoff E.T., Folta S.C., Anzman-Frasca S., Economos C.D. (2020). Orders of healthier adult menu items in a full-service restaurant chain with a healthier children’s menu. Nutrients.

[76] Gavahian M., Mousavi Khaneghah A., Lorenzo J.M., Munekata P.E.S., Garcia-Mantrana I., Collado M.C., Meléndez-Martínez A.J., Barba F.J. (2019). Health benefits of olive oil and its components: Impacts on gut microbiota antioxidant activities, and prevention of noncommunicable diseases. Trends Food Sci. Technol..

[77] Barilla Center for Food and Nutrition (2020). The Amazing Twelve: 12 Recommendations for a Healthy and Sustainable Diet. https://www.barillacfn.com/en/magazine/food-and-sustainability/12-recommendations-for-a-healthy-and-sustainable-diet/.

[78] Morawicki R.O., Díaz González D.J. (2018). Food sustainability in the context of human behavior. Yale J. Biol. Med..

