# Supplementary material for: Evaluating Mediterranean Diet-Adherent, Healthy and Allergen-Free Meals Offered in Tarragona Province Restaurants (Catalonia, Spain): A Cross-Sectional Study
Source: Nutrients. 2021 Jul 19;13(7):2464. doi: 10.3390/nu13072464 (PMC8308532; doi:10.3390/nu13072464)
Supplement: Supplementary file 1 [file nutrients-13-02464-s001.zip › nutrients-1289602-supplementary.pdf]

Table S1: STROBE checklist for observational cross-sectional studies.

	Item No	Recommendation	Page
**Title and abstract**	1	(*a*) Indicate the study's design with a commonly used term in the title or the abstract	1
		(*b*) Provide in the abstract an informative and balanced summary of what was done and what was found	1
		**Introduction**	
Background/rationale	2	Explain the scientific background and rationale for the investigation being reported	1-2
Objectives	3	State specific objectives, including any prespecified hypotheses	2
		**Methods**	
Study design	4	Present key elements of study design early in the paper	2-3
Setting	5	Describe the setting, locations, and relevant dates, including periods of recruitment, exposure, follow-up, and data collection	3
Participants	6	(*a*) Give the eligibility criteria, and the sources and methods of selection of participants	3
Variables	7	Clearly define all outcomes, exposures, predictors, potential confounders, and effect modifiers. Give diagnostic criteria, if applicable	3-5
Data sources/ measurement	8	For each variable of interest, give sources of data and details of methods of assessment (measurement). Describe comparability of assessment methods if there is more than one group	3-5
Bias	9	Describe any efforts to address potential sources of bias	-
Study size	10	Explain how the study size was arrived at	-
Quantitative variables	11	Explain how quantitative variables were handled in the analyses. If applicable, describe which groupings were chosen and why	
Statistical methods	12	(*a*) Describe all statistical methods, including those used to control for confounding	5
		(*b*) Describe any methods used to examine subgroups and interactions	5
		(*c*) Explain how missing data were addressed	-
		(*d*) If applicable, describe analytical methods taking account of sampling strategy	-
		(*e*) Describe any sensitivity analyses	-
		**Results**	
Participants	13	(a) Report numbers of individuals at each stage of study—eg numbers potentially eligible, examined for eligibility, confirmed eligible, included in the study, completing follow-up, and analysed	5-6
		(b) Give reasons for non-participation at each stage	5-6
		(c) Consider use of a flow diagram	6
Descriptive data	14	(a) Give characteristics of study participants (eg demographic, clinical, social) and information on exposures and potential confounders	6-9
		(b) Indicate number of participants with missing data for each variable of interest	-
Outcome data	15	Report numbers of outcome events or summary measures	9-14
Main results	16	(*a*) Give unadjusted estimates and, if applicable, confounder-adjusted estimates and their precision (eg, 95% confidence interval). Make clear which confounders were adjusted for and why they were included	9-14
		(*b*) Report category boundaries when continuous variables were categorized	-

		(*c*) If relevant, consider translating estimates of relative risk into absolute risk for a meaningful time period	-
Other analyses	17	Report other analyses done—eg analyses of subgroups and interactions, and sensitivity analyses	14
		**Discussion**	
Key results	18	Summarise key results with reference to study objectives	14-17
Limitations	19	Discuss limitations of the study, taking into account sources of potential bias or imprecision. Discuss both direction and magnitude of any potential bias	17
Interpretation	20	Give a cautious overall interpretation of results considering objectives, limitations, multiplicity of analyses, results from similar studies, and other relevant evidence	14-17
Generalisability	21	Discuss the generalisability (external validity) of the study results	16-17
		**Other information**	
Funding	22	Give the source of funding and the role of the funders for the present study and, if applicable, for the original study on which the present article is based	17

Table S2: Correlation analysis between total number of AMed criteria fulfilled and green-light dishes.

**Green-light dishes rated in the traffic light system per nutrient**	**Total number of AMed criteria fulfilled**	***p*-value**
Energy[1]	-.20	.41
Carbohydrates[2]	-.12	.44
Sugar[2]	.32	.04*
Protein[1]	-.32	.11
Total fat[2]	.57	.03*
Saturated fat[2]	.09	.61
Sodium[2]	-.11	.52
Fibre[2]	-.32	.03*

[1]: Pearson correlation coefficient (r); [2]: Spearman correlation coefficient (p); *: significant values at $p<.05$.

Table S3: Restaurants' purchased foods.

N=66 respondents<br>Where was made the food purchase, % (n)						
	**Fish[1]**	**Meat[1]**	**Fruit[1]**	**Vegetable[1]**		
Traditional stores	15.2 (10)	10.6 (7)	24.2 (16)	27.3 (18)		
Market	25.8 (17)	24.2 (16)	31.8 (21)	25.8 (17)		
Supermarket	13.6 (9)	7.6 (5)	10.6 (7)	10.6 (7)		
Door-to-door	15.2 (10)	10.6 (7)	10.6 (7)	15.2 (10)		
Wholesalers	78.8 (52)	78.8 (52)	66.7 (44)	63.6 (42)		
Cooperative	6.1 (4)	3.0 (2)	13.6 (9)	15.2 (10)		
Own production	4.5 (3)	1.5 (1)	7.6 (5)	6.1 (4)		
Other	4.5 (3)	4.5 (3)	4.5 (3)	6.1 (4)		
Not bought	4.5 (3)	4.5 (3)	3.0 (2)	4.5 (3)		
**N=66 respondents**						
**Kind of eggs purchased, % (n):**	**Ecologic[1]**	**Farm[1]**	**Free-range[1]**	**Cage[1]**	**Pasteurized[1]**	**Other[1]**
	24.2 (16)	36.4 (24)	15.2 (10)	21.2 (14)	27.3 (18)	7.6 (5)
**N=66 respondents**						
**Type of oil purchased and used, % (n):**	**Kind of oil purchased[1]**	**Raw seasonings and sauces[1]**	**Grilled, roasted foods[1]**	**Fried food[1]**	**Candied foods[1]**	
Extra virgin olive oil	84.8 (56)	90.9 (60)	46.9 (31)	16.6 (11)	39.4 (26)	
Virgin olive oil	22.7 (15)	10.6 (7)	24.2 (16)	3.0 (2)	16.6 (11)	
Olive oil	18.2 (12)	4.5 (3)	15.1 (10)	4.5 (3)	6.1 (4)	
Sunflower oil	57.6 (38)	27.1 (18)	16.5 (11)	39.2 (26)	10.5 (7)	
Other type of oil	22.7 (15)	9.0 (6)	7.5 (5)	28.7 (20)	4.5 (3)	

[1]: Responses were given by restaurateurs and cooks; the total percentage of respondents is higher than 100% due to the multiple-option responses given by restaurateurs and cooks.

Table S4: Recommendations for restaurants to increase Mediterranean menu offering and improve food allergen management.

**Recommendations for restaurants to increase Mediterranean menu offerings[1]:**	**Recommendations for restaurants to improve food allergen management[1]:**
**1)** Substitute refined-grains products such as pasta, pizza, rice, bread and other white-flour-based foods for whole-grains options, to increase the consumption of fibre which has been related to many beneficial health effects [73];	**1)** Provide more training courses to the kitchen and dining room's staff about the correct practices in case of allergic and intolerant customers, with both theorical and practical sessions;
**2)** Prefer the use of fresh fruits as naturally sweet ingredients for the preparation of desserts, to reduce the use of added sugar;	**2)** Improve the availability of kitchen tools for the exclusive cooking of allergen-free meals;
**3)** Reduce servings' portion sizes to cut on excessive energy content, and allow the choosing of menu dishes by children;	**3)** Identify the presence of food allergens on the menu, as well as serve more information to customers through menu indicators about the availability of healthier

	offerings, options for children, vegetarian and vegan meals.
**4)** Increase the offer of vegetarian and vegan meals, to meet the needs of customers who follow these special diets, but also to reduce the exceeding intake of calories, total fats, proteins and carbohydrates;	
**5)** Lower the use of salt by preferring the use of spices and aromatic herbs;	
**6)** Improve the promotion of existing healthy options, especially when limited resources can be invested in the development of new offerings, as is the case for most independent restaurants that operate with narrower profit margins [13].	
**Positive practices observed at the included restaurants:**	
**1)** The use of extra virgin olive oil for dressing and cooking, which is the most representative component of the Mediterranean diet and has been associated with many health positive effects due to the high content of bioactive compounds (e.g., polyphenols) [74];	
**2)** The offer of fresh seasonable and traditional local foods, which contribute to the sustainability of the diet by lowering the environmental impact due to the goods' packaging [75] and transport [76];	
**3)** Prioritize culinary preparations that do not require the addition of large amounts of fat, such as backing and roasting instead of frying, whose frequent consumption is correlated to different adverse health effects because of the nutrient's loss, the increase of trans-fatty acids, the development of thermal degradation and oxidation substances [77].	

[1]: Recommendations were developed according to the present cross-sectional analysis results.
